# Deep Learning-Based Indoor Air Quality Forecasting Framework for Indoor Subway Station Platforms

**DOI:** 10.3390/toxics10100557

**Published:** 2022-09-23

**Authors:** Ahtesham Bakht, Shambhavi Sharma, Duckshin Park, Hyunsoo Lee

**Affiliations:** 1 School of Industrial Engineering, Kumoh National Institute of Technology, Gumi 39177, Korea; 2 Transportation System Engineering, University of Science and Technology (UST), Daejeon 34113, Korea; 3 Department of Transportation Environmental Research, Korea Railroad Research Institute (KRRI), Uiwang 16105, Korea

**Keywords:** particulate matter, indoor subway station, deep learning, hybrid CNN-LSTM, ventilation control

## Abstract

Particulate matter (PM) of sizes less than 10 µm ($PM_{10}$) and 2.5 µm ($PM_{2.5}$) found in the environment is a major health concern. As PM is more prevalent in an enclosed environment, such as a subway station, this can have a negative impact on the health of commuters and staff. Therefore, it is essential to continuously monitor PM on underground subway platforms and control it using a subway ventilation control system. In order to operate the ventilation system in a predictive way, a credible prediction model for indoor air quality (IAQ) is proposed. While the existing deterministic methods require extensive calculations and domain knowledge, deep learning-based approaches showed good performance in recent studies. In this study, we develop an effective hybrid deep learning framework to forecast future $PM_{10}$ and $PM_{2.5}$ on a subway platform using past air quality data. This hybrid framework is an integration of several deep learning frameworks, namely, convolution neural network (CNN), long short-term memory (LSTM), and deep neural network (DNN), and is called hybrid CNN-LSTM-DNN; it has the characteristics to capture temporal patterns and informative characteristics from the indoor and outdoor air quality parameters compared with the standalone deep learning models. The effectiveness of the proposed $PM_{10}$ and $PM_{2.5}$ forecasting framework is demonstrated using comparisons with the different existing deep learning models.

## 1. Introduction

Subway transportation is operated globally to cope with rising ground traffic congestions. Fast and convenient subway transport systems help to reduce the traffic pressure within cities [1]. With more than 310 subway stations on ten lines, Seoul is one of the largest and busiest metropolitan cities. Each subway line carries about 700,000 passengers on weekdays and 300,000 passengers on weekends [2]. While it offers a convenient way of transportation, its internal air quality raises concern. If not properly ventilated, it causes nitrogen dioxide, carbon dioxide, carbon monoxide, and particulate matter to accumulate over time [3]. Particulate matter (PM) and pollutants such as sulfur dioxide ($SO_{2}$), nitrogen oxides ($NO_{x}$), carbon monoxide (*CO*), and others that are present in the air above a certain threshold are known to cause several health problems, such as non-malignant respiratory disease, asthma, and allergies; a higher mortality rate; and early death [4,5]. Particulate matter (PM) recently received much attention because of its negative health impacts. $PM_{2.5}$ and $PM_{10}$ have aerodynamic dimensions less than 2.5 µm ($PM_{2.5}$) and 10 µm ($PM_{10}$), which can erode the alveolar wall, decrease lung function, and induce various cardiovascular disorders [6,7,8]. Existing studies [9,10,11,12,13] have stated that the concentration of airborne particles in a subway station can be up to ten times higher than the recommended WHO exposure limit. Additionally, the increase in PM concentrations has several negative impacts on the economy [14,15]

Indoor air quality (IAQ) in subway stations depends on various factors, such as outdoor air quality, climatic conditions, abrasion during operations, passenger loads, and subway schedule [16,17]. Studies showed that outdoor $PM_{2.5}$ could filtrate indoor buildings even with closed doors [18]. Shrestha et al. [19], in their studies of 28 low-income homes in Denver, Colorado, during the 2016 and 2017 wildfire seasons, showed that outdoor air pollution related to traffic and wildfires increased the indoor air pollutant concentrations due to infiltration and natural ventilation. Other studies showed how the wildfires smoke transported by wind affects the quality, atmospheric chemistry, and visibility of places located hundreds of kilometers away from the location of wildfires [20,21]. Wang et al. [22], in their study, accounted that socio-economic factors such as industrial emissions (i.e., soot, $SO_{2}\;\mathrm{and}\;NO_{x}$), population density, foreign direct investment, and per capita GDP had significant influences on the environmental $PM_{2.5}$ concentrations.

A traditional mechanical ventilation system is commonly observed in subway stations for regulating interior pollutants. It plays an important role in reducing the particulate matter and the energy demand of the subway station [23]. However, its operating mechanism fails to account for the real-time fluctuation in the parameters that may cause energy waste or deficiency. Forecasting $PM_{2.5}$ and $PM_{10}\;$ concentrations on platforms is critical for establishing early warning systems and managing ventilation systems to maintain commuter safety [24,25]. In order to forecast these PMs, a new and effective hybrid deep learning framework is proposed. The newly devised framework shows better forecasting performance than existing forecasting frameworks, including contemporary deep learning machines.

The main contribution of this study includes the development of a hybrid CNN-LSTM-DNN framework; we compare its performance with that of existing state-of-the-art deep learning techniques, the RNN and its variants (LSTM and Bi-LSTM), the CNN, and the DNN. The comparison of the performance of each of the deep learning architectures was analyzed using the root mean square error (RMSE), the mean absolute error (MAE), and R^2^. The predictive monitoring of $PM_{10}$ and $PM_{2.5}$ can help to develop an early monitoring system and to control a ventilation system to maintain sustainable indoor air quality on subway platforms.

The remainder of this paper is organized as follows: In the following section, the relevant background and literature review are provided. Section 3 gives information about the availability of data, the correlations among input data variables, and the model description. In the next section, the analysis and the discussion of the results obtained using different DL frameworks are given. Lastly, the paper is concluded, highlighting the limitations of the present study and future directions.

## 2. Background and Literature Review

In order to forecast indoor air quality, the first step is to measure the number of contaminants in the air, which may be conducted by putting sensors in strategically placed sites [26]. Placing sensors in many of these sites can be expensive and unfeasible. An alternate strategy could be the use of mathematical models utilizing data obtained from sensors over an extended period and the prediction of their patterns using these models. As a result, there have been a lot of efforts in recent years to construct environmental models using different methodologies [27,28,29].

Commonly used methods for forecasting air pollutants can be categorized as mathematical, statistical, and machine learning methods. Mathematical models or deterministic methods require specific knowledge for parameter identification and know-how of the processes. To overcome the limitation of deterministic models, statistical models that require a large number of observed data were developed. Jian et al. [30] applied an auto-regressive integrated moving average (ARIMA) model to predict the submicron particle concentration in Hangzhou, China. Another stochastic ARIMA model by Slini et al. [31] was used to forecast ozone concentration in Athens, Greece. One drawback of these models is that they consider the relationship between the responses and predictors with comparatively simple linear models. At the same time, these models based on statistics are limited due to linear assumptions and ignorance of multicollinearity.

To overcome this issue, non-linear machine learning (ML) models [32], such as support vector machine [33], k-nearest neighbor [34], fuzzy logic [35], and artificial neural network models [36,37], were adopted. Goulier et al. [37] used an artificial neural network to predict the hourly NO_2_ concentration in Central London. However, these machine-learning-based methods are not fully capable of learning from long-term dependencies or capturing time-series patterns from IAQ data [38]. Conventional machine learning and shallow networks are no longer state-of-the-art techniques, as they are unfit to capture the dynamic behavior of PM. Contemporary artificial intelligence (AI) and deep learning techniques are evolved to describe the complex, nonlinear PM relationship in an IAQ system. With several advancements in the areas of deep learning, they can extract features by learning from a large number of data [39,40]. Various deep learning methods are widely applied in air quality monitoring and water effluent quality prediction [41]. The unique ability of deep learning approaches is to learn from the vast number of data without prior experience, and they have many advantages over classical algorithms.

Various deep learning approaches, including the deep recurrent neural network (RNN) and convolutional neural network (CNN), were developed and improved for performing tasks ranging from regression to classification, to prediction. Loy et al. [42] used several types of RNN (long short-term-memory, gated recurrent unit) structures to predict hourly $PM_{2.5}$ in a subway station in South Korea. Long short-term memory (LSTM), a variant of the RNN, stands out in time-series forecasting problems due to its property of long-term memory. CNN is a popular technique for image recognition and classification and is successfully applied for time-series forecasting tasks [43]. CNN and other deep learning models are widely used in real-time air quality modeling [44]. Shahzeb et al. [3] used a residual neural network (Resnet-50)-based modified version to predict $PM_{2.5}$ concentration in a newly built subway station. Its input data consisted of 5 input attributes and 12 past observations.

Shengdong et al. [45] proposed a hybrid deep learning framework for predicting air quality ($PM_{2.5}$) in Beijing, China. Rahmadani and Lee. [46] proposed a hybrid deep learning model with an LSTM model and ordinary differential equations to model the epidemic prediction framework of SARS-CoV-2. Lee et al. [47] proposed a real-time hybrid deep learning architecture using an RNN and a general DNN to predict running safety for a high-speed train. Yang et al. [48] proposed a model based on empirical mode decomposition and LSTM modules to forecast $PM_{2.5}$ in a subway platform. However, these methods are limited from the fact that detailed analyses and comparisons with existing deep learning models are provided comparatively less.

## 3. Hybrid CNN-LSTM Framework for Forecasting Indoor Subway Air Quality

### 3.1. Data and Preliminary Information

**Figure 1:**
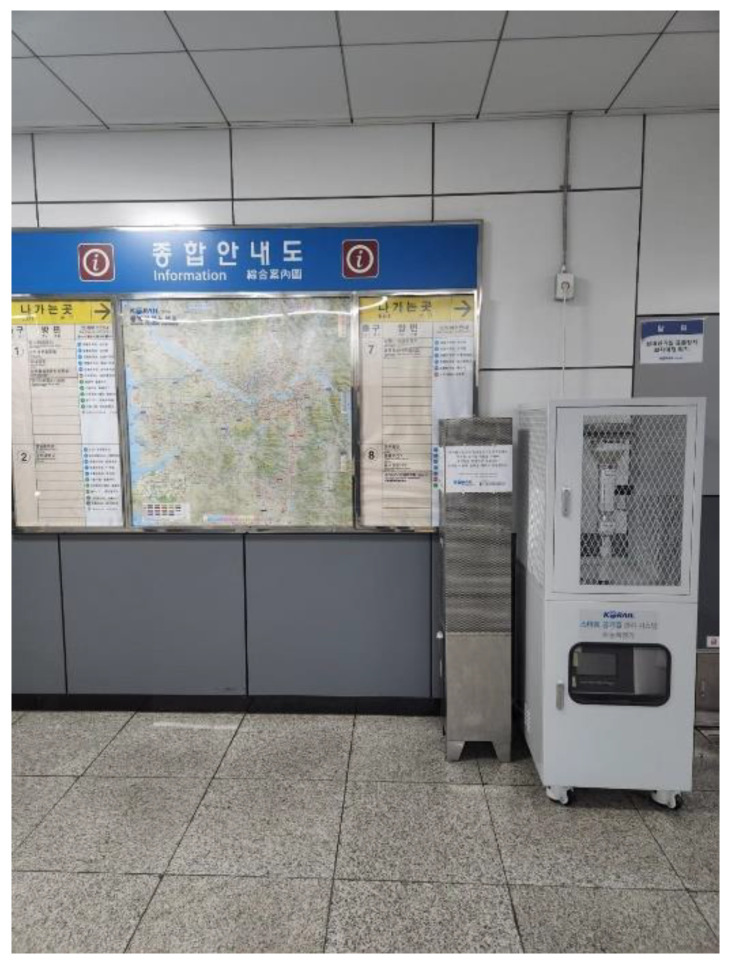
Spectrometer (Model 11-A) for detecting airborne particles at Yeongtong subway station.

In this investigation, measurements at the Yeongtong station were made using information from two separate sources. The ambient data were obtained from the Air-Korea website (www.inair.or.kr (accessed on 26 April 2022)), and a GRIMM aerosol spectrometer was used to detect particulate indoors. Figure 1 shows the tele-monitoring system (Model 11-A) used to collect the real-time PM concentration at the platform. The Model 11-A portable aerosol spectrometer detected airborne aerosol particles in the size range of 0.25 µm to 32 µm in 31 channels.

The platform of interest was on the second floor below the surface. The platform and the rail were fully sealed. The platform was the facing type, meaning persons wishing to go in one direction faced people who wished to go in the opposite direction. Subway trains ran from 5:15 am to 11:12 pm during weekdays and between 5:15 am and 12:17 am (the next day) during weekends. The average number of passengers travelling each day was 14,578 at the Yeongtong subway station. The flow of the passengers was not restricted due to COVID-19; however, masks were compulsory for travelling passengers during the study period. A PLC-based mechanical ventilation was used during operating hours. The efficiency of the ventilation system in removing the particulate matter was between 50 and 55% via capture-filtering using a medium filter.

**Figure 2:**
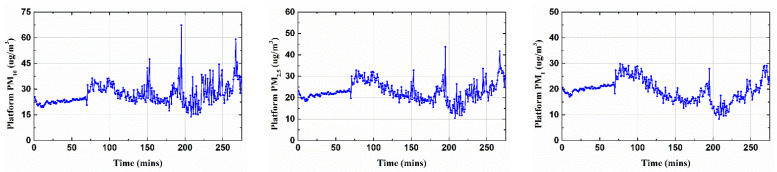

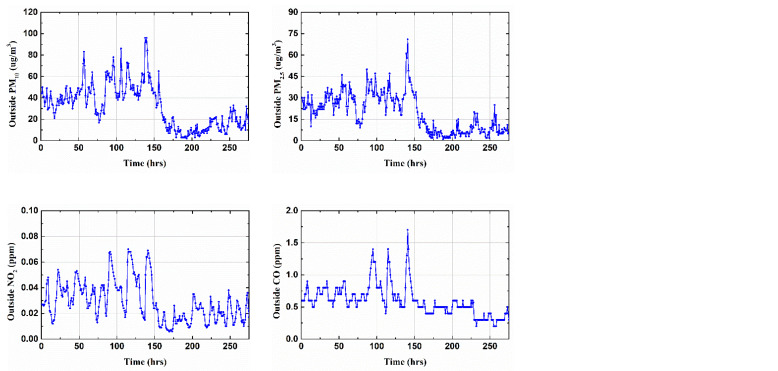
Variation in the input variables over time on the platform and outside the subway station.

This study considered the measurement of $PM_{10}$, $PM_{2.5}$ and $PM_{1}$ at the Yeongtong subway station from 22 October 2021 to 26 November 2021 and the measurement of $PM_{10}$, $PM_{2.5}$, $PM_{1}$, $NO_{2}$, and *CO* outside the subway station (within 500 m from the Yeongtong subway station) during the same period of time. The platform data were collected every six seconds. As a preprocessing step, the data were averaged to a 5 min interval for our analyses. Figure 2 shows the measurement trends of components both inside and outside the subway station.

**Figure 3:**
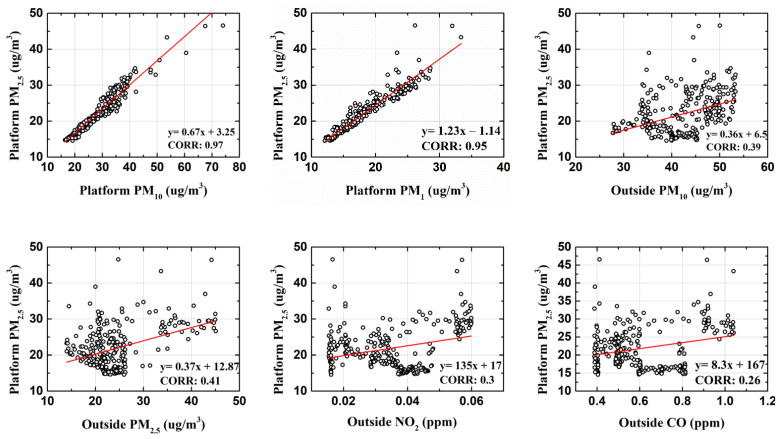
Correlation analysis of $PM_{2.5}$ with other measured variables.

Table 1 summarizes the basic statistics of the measured variables and data. Platform $PM_{10}$ and $PM_{2.5}$ were influenced by many inside and outside factors. A preliminary linear regression was performed to determine the correlation between the inside and outside variables. Figure 3 shows the correlation between platform $PM_{2.5}\;$ and the variables.

**Figure 4:**
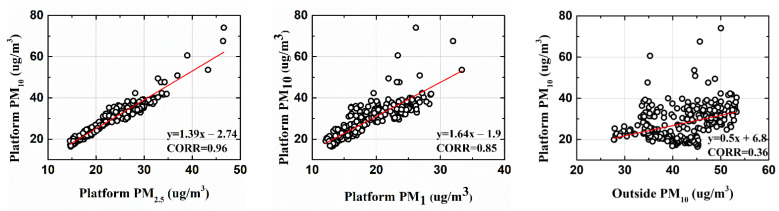

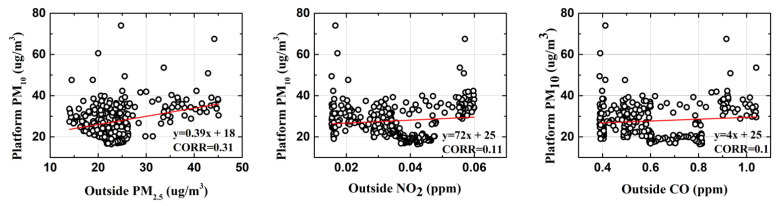
Correlation analysis of $PM_{10}$ with other measured variables.

As shown in Figure 3, platform $PM_{2.5}\;$ and platform $PM_{10}$ had a strong correlation. The information on *CO* and $NO_{2}$ indicated more vehicular emission; consequently, it depicted an implicit relation with particulate matter. Analogously, particulate matter from the outside may have also infiltrated the inside of the subway, which was indicated by the correlation values of 0.41 and 0.39. Those variables that showed very low coefficients of correlation (<0.1) were dropped, and only those with CORR values greater than 0.2 were considered for the forecast of platform $PM_{10}$ and $PM_{2.5}$. Similarly, the linear regression test for platform $PM_{10}$ and other variables is shown in Figure 4.

### 3.2. Preprocessing for Hybrid Deep Learning Framework

**Figure 5:**
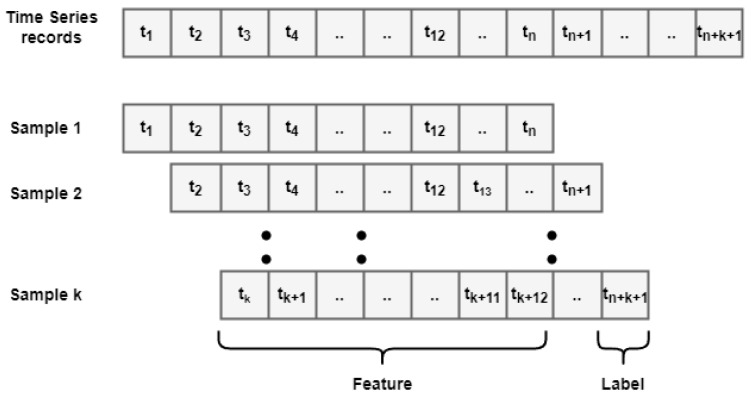
Time-series samples for the forecasting of platform $PM_{10}$ and $PM_{2.5}$.

The data that are mentioned in the above section were preprocessed to remove the missing values or outliers obtained due to the malfunction or shock of the sensors. The data obtained from the Yeongtong subway station is of six-second intervals. In order to integrate inner and outside signals, the time scale was modified to five-minute intervals. The outside station data were collected at a one-hour frequency. However, they were converted to five-minute-interval data using spline interpolation. The data were then transformed for the feasibility of the sequential temporal model. The sampling was obtained in the time period $\left[t_{n}-\Delta t,t_{n+k}\right]$, where $t_{n}$ is the current time in the *n^th^* sample; Δt is called the window size, and it refers to one hour in the past from the current time ($t_{n}$); $t_{n+k}$ is the ‘*k^th^*’ time ahead in the future. In this study, it was half an hour ahead in the future. Figure 5 shows the past input data (feature data) and the prediction target (the label data).

**Figure 6:**
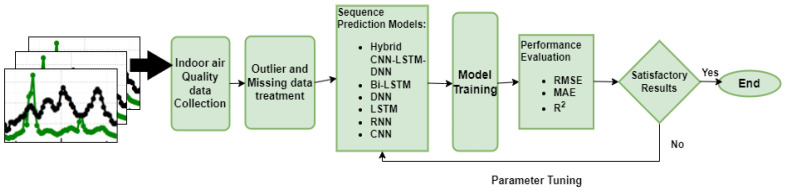
Workflow of the forecast of platform $PM_{10}$ and $PM_{2.5}$ on the Yeongtong subway platform using hybrid CNN-LSTM-DNN and other deep learning-based architectures.

As it can be seen, a larger window size (Δt) included more features and a smaller sample size, whereas a smaller window size gave more samples but fewer features. In the dataset, we had 7242 sample data points for training and 1080 sample data points for testing, collected over a period form 22 October to 26 November 2021 on the Yeongtong subway platform and outside. The forecasting workflow of platform $PM_{10}$ and $PM_{2.5}$ is given in Figure 6.

### 3.3. Proposed Hybrid Deep Learning Framework

**Figure 7:**
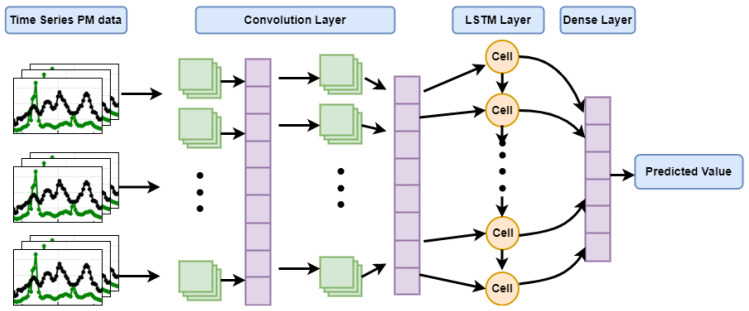
The structure of the proposed hybrid Conv-LSTM-DNN framework.

To build an efficient $PM_{10}$ and $PM_{2.5}$ prediction model, we propose a hybrid deep learning framework by integrating Conv1D with LSTM. Figure 7 shows the model structure of the proposed framework.

The proposed framework consists of an input layer, a convolution layer, an LSTM layer, a fully connected layer (DNN layer), and an output layer. The convolution layer learns the local features of the time-series sequence data using its convolutional operation. It shortens the length of time-series data and enhances the dependences among data. Each convolution layer has multiple filters, enabling it to learn more hidden features from the sequence data. The following LSTM block learns the long short-term dependencies in the sequence using the connection of memory cells. The subsequent fully connected layer maps the features into the sample space, while the output layer estimates the target PM value. The integration of the standalone framework with shared representation aids to build an effective time-series model that can learn intelligently from hybrid features. PM forecasting ($y_{pred}$) is denoted with function ‘f’ using nesting functions $F_{conv},\;\;F_{lstm},\;\;F_{fc}$ and the activation function, as shown in Equation (1).

(1) $$y_{pred}=f=F_{fc}(F_{lstm}(ReLU(BN(F_{conv}\left(X_{input}\right)))))$$

The forward propagation of the proposed deep learning framework follows the equations below.

(2) $$i_{t}=\sigma \left(W_{xi}*X_{t\;}+W_{hi}*{ℋ}_{t-1\;}+W_{ci}\circ \;C_{t-1\;}+b_{i}\right)$$

(3) $$f_{t}=\sigma \left(W_{xf}*X_{t\;}+W_{hf}*{ℋ}_{t-1\;}+W_{cf\;}\;\circ \;C_{t-1\;}+b_{f}\right)$$

(4) $$C_{t}=\left(f_{t}\;\;\circ \;C_{t-1\;}+i_{t\;}\;\circ \;tanh(W_{xc}*X_{t}+{ℋ}_{t-1\;}+b_{i}\right)$$

(5) $$o_{t}=\sigma \left(W_{xo}*X_{t\;}+W_{ho}*{ℋ}_{t-1\;}+W_{co}\;\circ \;C_{t\;}+b_{o}\right)$$

(6) $$ℋ=o_{t}\;\circ \;tanh\left(C_{t}\right)$$

where $X_{1}\ldots .X_{t}$ are all the inputs, the cell outputs are $C_{1}\ldots \ldots C_{t}$, and $H_{1}\ldots \ldots H_{t}$ are the hidden states of the proposed framework. ‘o’, denotes the Hadamard product, and ‘*’ is the convolutional operation. The discrepancy between the desired label, $‘y_{t}^{’}$, and the output, ‘$o_{t}$’, is evaluated using an objective function across all the ‘*T*’ time steps, as given in Equation (7).

(7) $$ℒ\left(x_{1},\ldots ,x_{t},y_{1},\ldots .y_{t},w_{h},w_{o}\right)=\frac{1}{T}\sum^{\;}l\left(y_{t},o_{t}\right)$$

As the backpropagation process, the gradient is computed with regard to the weight parameters, ‘*w*’, as shown in the equation below.

(8) $$\frac{\partial L}{\partial w_{h}}=\frac{1}{T}\overset{T}{\underset{t=1}{\sum }}\frac{\partial \left(y_{t},o_{t}\right)}{\partial w_{h}}\;$$

### 3.4. Comparisons with Existing Deep Learning Models

#### 3.4.1. LSTM and Bidirectional LSTM

LSTM is a special form of RNN architecture proposed by Hochreiter and Schmidhuber [49]. The traditional DNN fails to properly handle the time-series data, as input and output variables are assumed to be independent of each other. The LSTM network is selected owing to its ability to learn short and long impacts from historical air quality data. It shows good performance in air quality prediction [50,51]. LSTM is capable of handling arbitrarily long sequences. Bidirectional LSTM is an upgraded version of LSTM given by Graves and Schmidhuber [52]. For the modeling process, it also considers the information in later time series. In order to show the effectiveness of the proposed framework, the prediction was compared with that obtained using LSTM and Bidirectional LSTM.

#### 3.4.2. DNN and CNN

The DNN is a deep learning-based structure consisting of an input layer, hidden layers, and an output layer. The number of hidden layers is set by the user, and their main function is to transmit data from the input layer to the output layer. After the feed-forward step, the weights of each of the hidden layers are updated based on learning algorithms. We adopted ‘stochastic gradient descent’ for backpropagation. The parameters of this model, such as the number of hidden layers, learning rate, and momentum constant, were determined experimentally with the data. The used activation function was *tanh* with a dropout probability of 0.3, to prevent it from overfitting. The equations of the DNN were as shown below.

(9) $$z_{i}^{l}=\underset{i}{\sum }w_{i,j}^{l}*x_{i}^{l-1}+b_{j}^{l}$$

(10) $$a^{l}=tanh\left(z_{i}^{l}\right)$$

where ‘*w*’ is the weight matrix, ‘*x*’ is the input vector, and ‘*b*’ is the bias.

As another comparison model, the CNN is successfully used in image classification and, more recently, in multivariate time-series data. It is capable of automatically extracting partial features from the data using the convolution operation. Convolutional computing was calculated as shown below.

(11) $$y_{j}^{l}=\underset{i}{\sum }\left[x_{i}^{l-1}*{w^{l}}_{i,j}+b_{j}^{l}\right]$$

(12) $$x_{j}^{l}=ReLU\left(BN\left(y_{j}^{l}\right)\right)$$

(13) $$x_{k}^{l+1}=FC\left(w_{k,j}^{l+1}*x_{j}^{l}+b_{k}^{l+1}\right)$$

where * refers to the convolution operation, and $w_{I,j}^{l}$ and $b_{j}^{l}$ are the weights of filters and biases. $x_{i}^{l-1}$ and $y_{j}^{l}$ represent the input and the output of the ‘l’ convolution layer. Each convolution layer is followed by a batch normalization and *ReLU* activation function.

## 4. Indoor Air Quality Forecasting and Comparison Analysis

In order to compare the forecasting performance, the RMSE (root mean square error), the MAE (mean absolute error), and $R^{2}$ (coefficient of determination) were considered and were calculated using Equations (14)–(16), where $y_{true}^{i}\;and\;y_{pred}^{i}$ are the true and predicted values, $\bar{y}$ is the average of the truth data, and *‘m’* is the number of test samples.

(14) $$RMSE=\sqrt{\frac{1}{m}\overset{m}{\underset{i=1}{\sum }}{\left(y_{pred}^{i}-y_{true}^{i}\right)}^{2}}$$

(15) $$MAE=\frac{1}{m}\overset{m}{\underset{i=1}{\sum }}|y_{pred}^{i}-y_{true}^{i}|$$

(16) $$R^{2}=1-\frac{\sum_{i=1}^{m}{\left(y_{pred}^{i}-y_{true}^{i}\right)}^{2}}{\;\sum_{i=1}^{m}{\left(\bar{y}-y_{true}^{i}\right)}^{2}}$$

**Figure 8:**
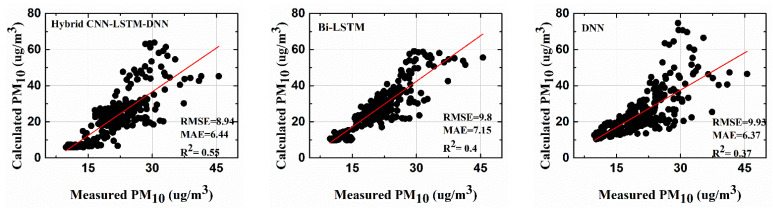

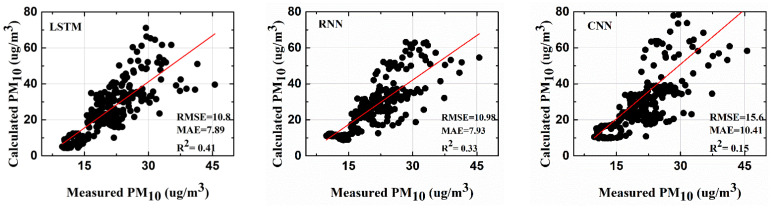
R^2^ comparisons for the calculated platform $PM_{10}$ and measured platform $PM_{10}$.

In this section, the performance of each mentioned stand-alone architecture is compared with that of the proposed framework (hybrid CNN-LSTM-DNN framework). For the comparisons, $PM_{10}$ and $PM_{2.5}\;$ at the platform were forecasted on the time scale of thirty minutes ahead. Then, past data of an hour from the target time were used to predict $PM_{10}$, and $PM_{2.5}$ thirty minutes ahead. As explained in Section 3, the past data were averaged at five-minute intervals, giving twelve attributes for each of the input variables. The performance of each of the deep learning models was evaluated using the RMSE, the MAE, and R^2^. Figure 8 shows the calculated and the measured $PM_{10}\;$ values for the Yeongtong subway platform using different deep learning architectures. The prediction models were implemented using Matlab^®^ 2021Rb.

The results showed the superior performance of the proposed hybrid deep learning framework in terms of all the performance metrics (RMSE, MAE, and R^2^) as compared with the other standalone deep learning architectures. The prediction accuracy for platform $PM_{10}$ was the highest in the case of the hybrid CNN-LSTM-DNN framework, as depicted by the highest R^2^, 0.55, and the lowest RMSE and MAE values, 8.94 and 6.44, respectively (as shown in Table 2). Bidirectional LSTM performed well in the prediction of both platform $PM_{10}$ and $PM_{2.5}$, with RMSE values of 9.8 and 11.95, respectively. The performance of the DNN with regard to the RMSE was good for platform $PM_{10}$ but not so good for platform $PM_{2.5}.$

**Figure 9:**
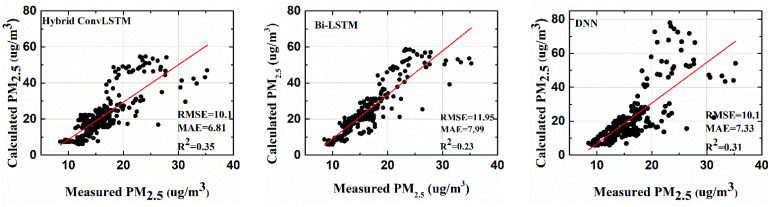

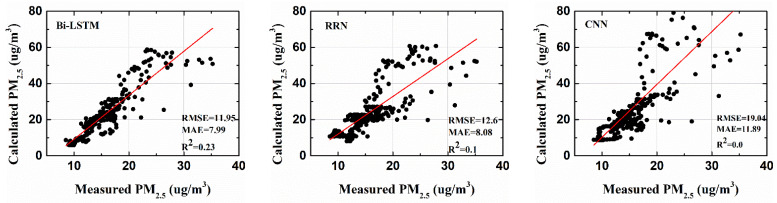
R^2^ comparisons for the calculated platform $PM_{2.5}$ and measured platform $PM_{2.5}$.

A similar forecasting performance for the estimated platform $PM_{2.5}\;$ and the measured platform $PM_{2.5}$ is given in Figure 9.

**Figure 10:**
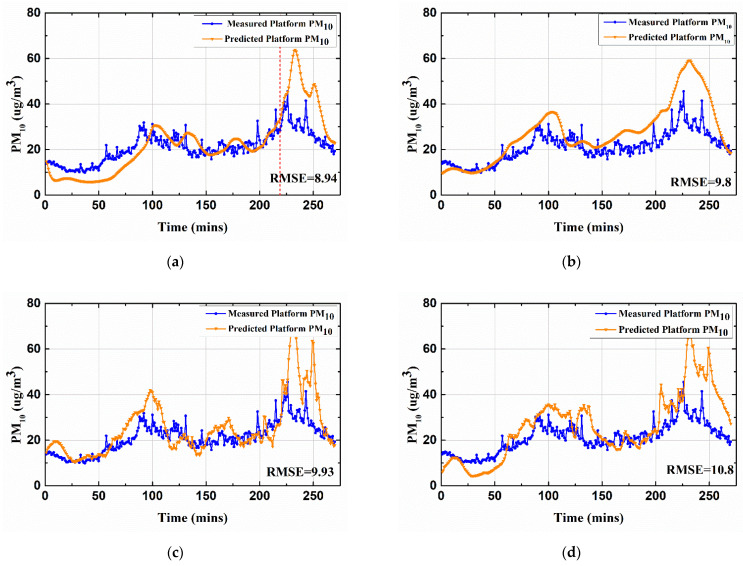

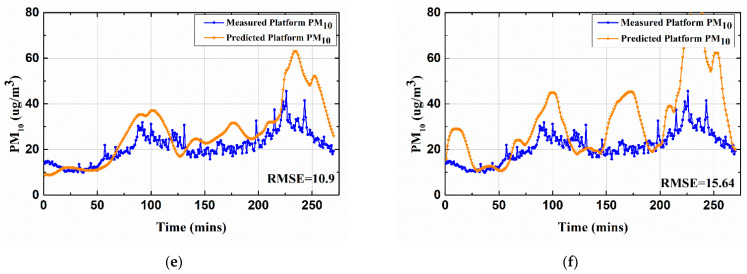
Half-an-hour-ahead forecasting results for platform $PM_{10}$ for different deep learning models. (**a**) Hybrid deep learning framework (the proposed model), (**b**) BiLSTM, (**c**) DNN, (**d**) LSTM, (**e**) RNN, and (**f**) CNN.

Figure 10 shows the overall forecasting and the RMSE measures.

**Figure 11:**
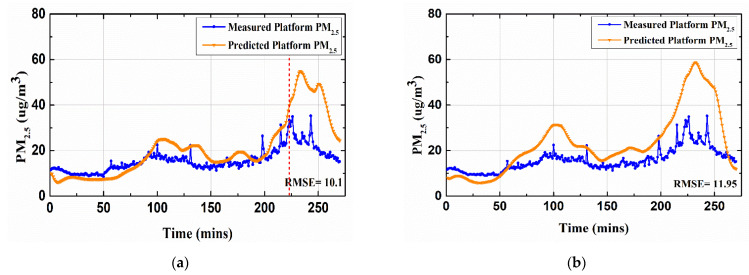

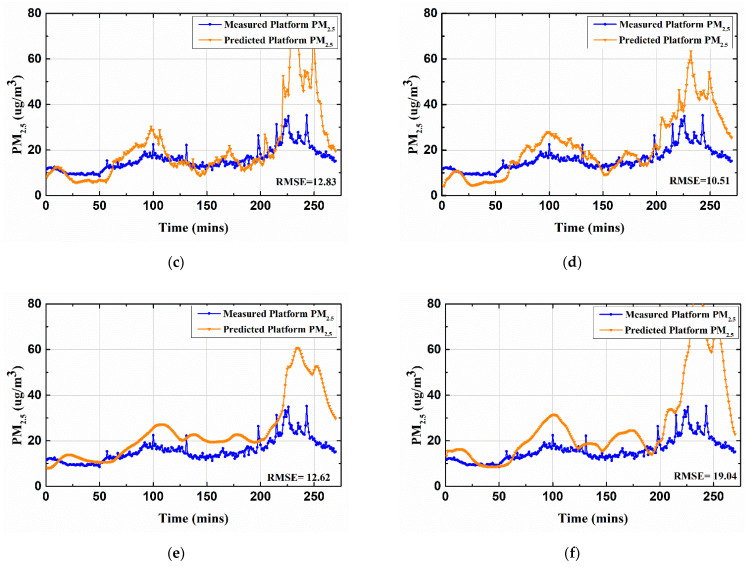
Half-an-hour-ahead forecasting results for platform $PM_{2.5}$ using different deep learning models. (**a**) Hybrid deep learning framework (the proposed model), (**b**) BiLSTM, (**c**) DNN, (**d**) LSTM, (**e**) RNN, and (**f**) CNN.

The variation pattern obtained showed that the forecasted data and the actual measurements were close when using the proposed hybrid deep learning framework. However, a little more variation in the measurements of platform $PM_{10}$ was observed for all the models during peak hours (after the 220th data point), as shown with a red vertical line in Figure 10a. This variation in fluctuation was not very high for the hybrid deep learning framework as compared with the other frameworks. The RMSE and MAE for the prediction of platform $PM_{10}$ were improved by 8.7% and 10% compared with the second-best deep learning framework, Bi-LSTM. Similarly, for the prediction of platform $PM_{2.5}$, the RMSE and MAE improved by 4% and 10%, respectively, with respect to the second-best deep learning-based framework, LSTM. It could be concluded that the proposed hybrid framework was well able to mimic the behavior of the measured platform $PM_{10}$. Thus, the estimated value of the forecasted platform $PM_{10}\;$ served as a precursor to the incoming peak in the measured value. A similar trend was also observed for the comparison of the measured $PM_{2.5}$ and the predicted platform $PM_{2.5}$, as shown in Figure 11.

## 5. Conclusions

The main highlights of this study are the integration of several deep learning methods into one, called hybrid CNN-LSTM-DNN framework, to make a prediction of $PM_{10}$ and $PM_{2.5}$. The performance of the proposed model in terms of forecasting $PM_{10}$ and $PM_{2.5}\;$ was better than that of the reference models owing to its ability to capture temporal patterns and informative characteristics from the indoor and outdoor air quality parameters. The proposed hybrid deep learning framework yielded the best results, with an RMSE value of 8.94 and an MAE of 6.4.

The main contribution of this paper can be summarized as follows: The one-dimensional convolution operation filtered original sequence data and reduced their dimension. LSTM learned the long short-term dependencies and effectively built a predictive model. The proposed methodology highlighted the effectiveness of deep learning algorithms in treating the nonlinear, non-stationary time-series data for PM monitoring. A demonstration of the effectiveness of the proposed model was conducted by comparing it with other state-of-the-art deep learning techniques for forecasting platform $PM_{10}$ and $PM_{2.5}$. The forecasting of future platform $PM_{10}$ and $PM_{2.5}$ could be used as a reference variable for the control system of subway ventilation, since there is a time delay to reduce the current PM levels in the air. This could help to more effectively protect passengers from harmful exposure to particulate matter. In other words, the predictive monitoring of $PM_{10}$ and $PM_{2.5}$ could help to develop early monitoring systems and regulate ventilation systems to maintain a sustainable indoor air quality index.

This paper could be further improved by incorporating more data, for example, geographical and meteorological data such as temperature, humidity, wind speed and direction, etc. It is expected that the addition of such factors could improve the forecasting performance of the proposed model. Lastly, the effectiveness of the model needs to be explored in case of scant data or sensor failure. Future studies should take into consideration all the issues listed above to develop a robust model for the prediction of platform $PM_{10}$ and $PM_{2.5}.$

## Figures and Tables

**Table 1:** Basic statistics of the measured variables at the Yeongtong subway station and outside (22 October 26 to November 2021).

Item	$\mathrm{Platform}\;PM_{10}$	$\mathrm{Platform}\;PM_{2.5}$	$\mathrm{Platform}\;PM_{1}$	$\mathrm{Outside}\;PM_{10}$	$\mathrm{Outside}\;PM_{2.5}$	$\mathrm{Outside}\;NO_{2}$	Outside CO
	(µg/m^3^)	(µg/m^3^)	(µg/m^3^)	(µg/m^3^)	(µg/m^3^)	(ppm)	(ppm)
Minimum	1.93	1.89	1.27	1.98	0.90	0.01	0.19
Maximum	260.24	145.97	126.36	184.64	114.83	0.08	1.70
Mean	32.95	26.95	22.37	43.86	24.42	0.03	0.62
Standard Deviation	23.51	20.90	18.54	26.65	18.13	0.01	0.24

**Table 2:** Forecasting performance for platform $PM_{10}$ and $PM_{2.5}$.

Comparison Model	$\mathrm{Platform}\;PM_{10}$			$\mathrm{Platform}\;PM_{2.5}$		
	*RMSE*	*MAE*	*R^2^*	*RMSE*	*MAE*	*R^2^*
Hybrid Deep learning framework (proposed)	8.94	6.44	0.55	10.1	6.81	0.35
*BILSTM*	9.8	7.15	0.4	11.95	7.99	0.23
*DNN*	9.93	6.37	0.37	12.83	7.33	0.31
*LSTM*	10.8	7.89	0.41	10.51	7.55	0.34
*RNN*	10.98	7.93	0.33	12.62	8.08	0.1
*CNN*	15.64	10.41	0.15	19.04	11.89	0

## Data Availability

The data presented in this study are available from the author upon reasonable request.
